# The Toner Lectures

**Published:** 1874-01

**Authors:** 


*The Toner Lectures,* instituted to encourage the discovery of new truths for the advancement of medicine. Lectnre No. 1. *On the structure of cancerous tumors and the mode in which adjacent parts are invaded,* by J. J. Woodward, Assistant Surgeon United States Army, delivered March 28, 1872.

The above is one of the most interesting lectures it was ever our pleasure to read—replete with scientific facts, most scientifically handled. We sincerely wish all our readers possessed a copy. Dr. Toner, with a warmth and generosity of heart unusual in this close, selfish age, has made the profession his debtor largely beyond its ability to repay him, save in sincere admiration and gratitude. Dr. Woodward has, by this lecture, at a bound, placed his name among the great medical thinkers of the age. W. T. G.

